# Effects of fattening strategies on carcass traits, meat quality, fatty acid composition, and oxidative stability of *longissimus* muscle in yaks (*Bos grunniens*)

**DOI:** 10.1016/j.fochx.2026.103540

**Published:** 2026-01-14

**Authors:** Zhiyuan Ma, Hongshan Liu, Abraham Allan Degen, Lintong Zhang, Jiandui Mi, Jianwei Zhou

**Affiliations:** a State Key Laboratory of Herbage Improvement and Grassland Agro-Ecosystems, College of Pastoral Agriculture Science and Technology, Lanzhou University, Lanzhou 730000, China; b Gulang County Vocational Secondary Specialized School, Wuwei 733100, China; c Desert Animal Adaptations and Husbandry, Wyler Department of Dryland Agriculture, Blaustein Institutes for Desert Research, Ben-Gurion University of the Negev, Beer Sheva 8410500, Israel; d State Key Laboratory of Animal Disease Control and Prevention, College of Veterinary Medicine, Lanzhou University, Lanzhou 730000, China.

**Keywords:** Yak meat, Fattening strategies, Carcass traits, Myofiber, Nutritional value, Fatty acids, Oxidative stability

## Abstract

In the context of coexisting traditional and intensive yak production systems, clarifying the effects of different fattening strategies is crucial for optimizing meat quality and meeting consumer demands. This study compared three feeding regimes: traditional grazing (TG), low-concentrate (LC), and high-concentrate (HC) stall-feeding, in 18 yaks. The HC regimen enhanced carcass traits, average daily gain, dressing percentage, and marbling score, while also increasing myofiber size and drip loss. Shear force of meat from TG yak was greater than the two stall-fed groups and from LC yaks was greater than the HC yaks. Nutritionally, TG and HC meat had a higher polyunsaturated fatty acid (PUFA) content and PUFA:SFA ratio than LC meat. Importantly, grazing enriched n-3 PUFAs and improved oxidative stability, indicated by higher superoxide dismutase activity and lower malondialdehyde. The HC diet, however, increased n-6 PUFA and intramuscular fat. In conclusion, while feedlot finishing improves production efficiency and tenderness, grazing yields superior nutritional benefits and oxidative stability in yak meat.

## Highlights

• Feedlot-fed improves yak carcass traits, while grazing improves meat oxidative stability.
• High-concentrate diet enhances growth performance and improves meat quality in yaks.
• Grazing yaks produced meat with higher n-3 PUFAs (EPA, DHA) and a superior n-6/n-3 ratio.
• Diversified yak fattening strategies meet consumer demands for both quality and nutrition.

## Graphical abstract

**Figure:**
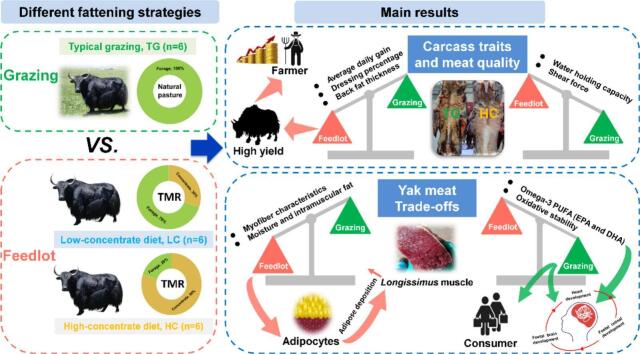

## 1 Introduction

The yak (*Bos grunniens*) is raised on grasslands on the Qinghai-Tibetan Plateau (QTP), at elevations between 3000 and 6000 m above sea level (Long et al., 2008). Historical records indicate that yak domestication was initiated by the ancient Qiang people approximately 7300 years ago (Qiu et al., 2012). Yaks serve as a cultural symbol and are indispensable to the livelihood of the local pastoral inhabitants, providing meat and milk for sustenance, hair and hides for textiles and garments, dung for fuel, and are used for transportation (Long et al., 1999; Luo et al., 2018). Traditionally, yaks were raised solely on natural grasslands, without supplements, even during winters (Zhou et al., 2017). Due to the harsh environment and diverse plant species on the QTP, meat from grazing yaks is considered organic and healthy, accounting for over 50% of the daily meat consumption of local Tibetan herders (Bai et al., 2009; Wu et al., 2007). Often referred to as the “crown of beef”, the meat is known for its high protein, low-fat content, and abundant amino acids, carotene, and other trace elements; however, the meat is considered tough (He & Feng, 2004; Lang et al., 2016; Zhang et al., 2023).

In recent years, the traditional grazing system has faced increasing challenges due to the growing yak population, ecological protection efforts, and pastoralists' pursuit of economic benefits, as natural pastures alone can no longer sustain yak rearing. Consequently, alternative yak-raising methods are being explored, with feedlots emerging as a viable solution to ensure the sustainability of yak production. Numerous feedlots have been established, primarily utilizing concentrate-based diets to promote rapid growth within a short timeframe. Several studies reported that yaks fed concentrates improved growth performance, meat yield, and meat quality (Kang et al., 2020; Liu et al., 2021). However, the negative aspects of feedlots, particularly concerning animal health and welfare, were often overlooked. For instance, high-concentrate diets can increase intramuscular fat content and accelerate weight gain but can also cause nutritional and metabolic disorders, such as rumen acidosis, due to low pH and high osmolality, and systemic inflammatory responses (Devries et al., 2014). Additionally, the rising costs of livestock feed components, such as corn and soybean meal, have placed financial pressure on high-concentrate fattening systems. In contrast, low-concentrate fattening, despite the slower growth rate, is gaining popularity due to the lower feeding costs and compatibility with traditional grazing practices. An optimal ratio of concentrates to forage in the diet can enhance animal performance and economic efficiency, particularly in a diversified yak fattening system.

With the global emphasis on healthy, green, and nutritious food, consumers are increasingly prioritizing meat quality, which influences purchasing decisions (Ortega et al., 2016). Carcass traits and meat quality are critical factors in the beef industry, with meat quality playing a substantial role in consumer preferences (Grunert et al., 2004). While feedlot rearing can maximize body fat deposition in the animal, it can also reduce the biosynthesis of unsaturated fatty acids (Zhang et al., 2022), potentially disrupting the oxidative balance within the meat (Cherif et al., 2018). However, a critical knowledge gap remains. Previous comparisons between “grazing” and “feedlot” systems often treated the latter as a homogeneous entity. A more nuanced understanding is needed, particularly regarding the impact of the concentrate level within feedlot systems. The distinction between low-concentrate (LC) and high-concentrate (HC) diets is crucial, as it represents a fundamental trade-off among growth performance, feeding costs, animal health, and ultimately, meat quality parameters. A systematic comparison of traditional grazing (TG) with both LC and HC feedlot regimens is essential to provide applicable insights for the yak industry. Consequently, this study was designed to comprehensively evaluate the effects of these three distinct fattening strategies (TG, LC, and HC) on a suite of quality attributes in yak *longissimus* muscle, encompassing carcass traits, meat physical quality, myofiber characteristics, detailed fatty acid composition, and oxidative stability markers.

## 2 Materials and methods

### 2.1 Study site, animal ethics, experimental design, and diets

The study was conducted from June to September at Wushaoling Yak Research Facility of Lanzhou University (37°12.4′ N, 102°51.7′ E, altitude 3154 m a. s. l.), Tianzhu Tibetan Autonomous County, Wuwei City, Gansu Province, P.R. China. All experimental protocols on the yaks were approved by the Animal Ethics Committee of Lanzhou University (protocol approval number: 20210520) and were conducted in accordance with the NIH (National Research Council) Guide for the Care and Use of Laboratory Animals.

Eighteen healthy, castrated male yaks (initial body weight, 216 ± 3.9 kg), aged 4 years old, were allocated randomly into three fattening treatments. One group grazed natural pasture without supplements as traditional management (TG, n = 6), while the other two groups were fed in a feedlot and offered a low-concentrate (LC, ∼ 30%, n = 6) and high-concentrate (HC, ∼ 80%, n = 6) diet. The number of yaks was based on the effect size index (d-value) which was calculated using estimated standard deviations of the means of measured variables from previous similar studies. A sample size of 6 per group resulted in a d-value close to 0.5, which is a medium and acceptable effect size (Sullivan & Feinn, 2012). Throughout the trial period (June to September), corresponding to the warm season of peak pasture productivity, TG yaks were managed under a continuous grazing system on a designated alpine meadow pasture without fencing and with free access to water. The dominant forage species of the grazed pasture was *Kobresia humilis*, but also included *Plantago depressa, Polygonum viviparum, Elymus nutans* and *Potentilla anserina,* with an average above-ground plant biomass of 49.0 g/m^2^ during the grazing period.

The LC and HC yaks were penned (1.2 m × 2.2 m) individually, and offered a total mixed rations (TMR) twice daily, at 06:30 and 18:00, and had free access to fresh drinking water. To maintain *ad libitum* feeding condition, at least 10% of feed remained daily. The experiment consisted of 10 days for adaptation followed by 90 days for measurements. The detailed ingredients and chemical composition of the pasture and TMR are presented in Table 1. The concentrate feed had greater concentrations of oleic acid (C18:1n9c) and linoleic acid (C18:2n6c) than the forage, while the forage had greater concentrations of palmitic acid (C16:0) and α-linolenic acid (C18:3n3) than the concentrate.

**Table 1:** Ingredients and chemical compositions of the diets of three yak groups.

Items	Experimental diets 1		
	TG 2	LC	HC
Ingredients, g/kg DM			
Oat hay	–	700	80.0
Wheat straw		–	120
Corn	–	120	320
Soybean meal	–	27.0	72.0
Cottonseed meal	–	36.0	96.0
Corn DDGS	–	25.0	68.0
Wheat bran	–	22.0	58.0
Distiller's grains	–	12.0	32.0
Corn germ meal	–	15.0	40.0
Sprayed corn bran	–	21.0	56.0
Soybean oil	–	3.00	8.00
Limestone	–	6.00	17.0
NaCl	–	3.00	8.00
NaHCO_3_	–	5.00	12.0
Urea	–	2.00	5.00
Premix 3	–	3.00	8.00
Chemical compositions 4, g/kg DM			
Dry matter	938	944	942
Crude protein	149	151	172
Ether extract	42	34	69
Neutral detergent fiber	538	442	292
Acid detergent fiber	268	270	159
Ash	72	110	97
Metabolizable energy, MJ/kg	9.4	9.7	12.7
Fatty acid, g/kg feed DM			
C12:0	0.59	0.08	0.06
C14:0	0.63	0.24	0.18
C16:0	5.85	3.18	3.96
C18:0	0.97	1.02	1.06
C18:1n9c	0.76	3.57	4.04
C18:2n6c	2.29	8.03	9.13
C18:3n3	8.82	0.45	0.54
C20:0	0.56	0.24	0.21
C22:0	0.73	0.28	0.23
C24:0	0.39	0.17	0.14

1 TG, traditional grazing; LC, low-concentrate stall-fed diet; HC, high-concentrate stall-fed diet.

2 The dominant plant species of pasture were *Kobresia humilis*, whereas the associated species were *Plantago depressa*, *Polygonum viviparum*, *Elymus nutans* and *Potentilla anserina*.

3 The premix provided the following as per kilogram: Fe, 4000 mg; Zn, 5000 mg; Cu, 600 mg; Mn, 2500 mg; Se, 50 mg; Co, 40 mg; I, 50 mg; vitamin A, 800,000 IU; vitamin D, 500,000 IU; vitamin E, 10,000 IU.

4 The metabolizable energy content was calculated according to the Tables of Feed Composition and Nutritive Values in China (Chinese Feed Database, 2021), while the other chemical composition values were measured.

### 2.2 Slaughter procedure and meat samples collection

At the end of the feeding period and following a 12-h fasting period, the yaks were slaughtered humanely in a commercial abattoir (Tianzhu Tibetan Autonomous County, Gansu, China) according to standard procedures of the Chinese beef industry (GB/T 19477–2018). The yaks were stunned by a captive-bolt pistol and exsanguinated by severance of the blood vessels in the neck. Following standard dressing procedures, the head, hide, legs, tail, viscera, diaphragm, and excess internal fat were removed, the carcass was split along the spine, and the hot carcass weight (HCW) was recorded.

*Longissimus* muscle (LM) samples were excised from the left carcass side between the 12^th^ and 13^th^ ribs. Subsamples were immediately flash-frozen in liquid nitrogen for oxidative stability analysis. The other subsamples were maintained at 4 °C for organoleptic quality assessment, and were then lyophilized and homogenized for chemical composition determination and fatty acid profiling.

### 2.3 Carcass measurements

Average daily gain (ADG) was calculated from the total weight gain/90 days measurement period. Dressing percentage (DP) was calculated as: hot carcass weight (HCW)/ final body weight (FBW) × 100 (Ornaghi et al., 2020). Subcutaneous back fat thickness was measured at the 12^th^ rib using a Vernier caliper (Shanghai Measuring Tools and Cutting Tools Factory Co., Ltd., Shanghai, China) following LM cross-sectioning. *Longissimus* muscle area (LMA) was determined by tracing a transverse cut between the 12^th^ and 13^th^ ribs onto sulfuric acid tracing paper. Marbling score was ranked from 1 to 5, by 10 trained observers, where 1 = no marbling and 5 = excessively abundant marbling, and the average score was used.

### 2.4 pH and meat colour

The pH of the LM was measured at 45 min and 24 h post-mortem using a portable pH meter (Testo 205, Testo AG, Lenzkirch, Germany). Prior to measurements, the electrode was calibrated with certified standard buffers (pH 4.0 and 7.0, at 25 °C). The integrated automatic temperature compensation system ensured measurement accuracy across the physiological temperature range. The electrode was inserted perpendicularly into the center of the muscle, and triplicate measurements were averaged.

Meat colour parameters, including lightness (L*), redness (a*), and yellowness (b*), were assessed using a calibrated chroma meter (FRU WR-18, Shenzhen Wave Optoelectronics Technology Co., Ltd., Shenzhen, China) with D65 illuminant, 10° observer angle, and 8 mm aperture. Following calibration by a white tile, three measurements per sample were taken after 30 min bloom time. Colour saturation (*C∗*) and hue angle (*H∗*) were calculated based on the L*, a*, and b* values (AMSA, American Meat Science Association, 2012).

### 2.5 Water holding capacity and shear force

The water-holding capacity (WHC) of the LM was assessed through drip loss, cooking loss, and pressing loss measurements. Drip loss and cooking loss were determined in triplicate samples following Honikel (1998) and the mean values were used. In brief, drip loss was determined in cylindrical muscle cores parallel to the muscle fiber orientation. Each core was weighed (initial weight, W₁), suspended by a sterile nylon thread in an individual polyethylene bag (to prevent surface contact), and stored at 4 °C for 24 h. After storage, samples were blotted gently with filter paper to remove surface fluids and reweighed (final weight, W₂). Drip loss percentage was calculated as: [(W₁ - W₂)/W₁] × 100. For cooking loss, the LM was weighed, wrapped in aluminum foil, and cooked in a preheated water bath until the internal temperature reached 70 °C (monitored with a calibrated thermocouple; Eirelec Ltd.™, Ireland). Samples were then cooled to 25 °C, dried, and reweighed, and cooking loss percent was calculated as:

$$\mathrm{Cooking loss}\left(\%\right)=\frac{\mathrm{Weight before cooking}-\mathrm{Weight after cooking}}{\mathrm{Weight before cooking}}*100$$

Shear force was measured using the cooked LM samples with a meat tenderness tester (RH-N50, Guangzhou Runhu Instruments Co., Ltd., Guangzhou, China). Each cooked LM sample was cut into six cylindrical cores parallel to the myofiber orientation using a round sampler (1.27 cm diameter). The average peak shear force, expressed in newtons (N), was used.

Pressing loss was determined using a 10 g LM sample with a pressure instrument (RH-1000, Guangzhou Runhu Instruments Co., Ltd., Guangzhou, China) following Prieto et al. (2008). Each sample was measured in triplicate, and the mean value is reported.

### 2.6 Histological characteristics

Within 2 h after slaughter, a small piece of muscle tissue (approximately 2.0 cm × 2.0 cm × 2.0 cm) was excised from the LM muscle and immersed immediately in a 10% formaldehyde solution. The samples were stored at 4 °C, and the fixation solution was replaced with fresh formaldehyde after 24 h. Subsequently, the tissue samples were paraffin-embedded, sectioned into slices, and stained with hematoxylin and eosin (H&E) for histological evaluation of muscle structure.

Under microscopic examination (Eclipse Ci-L, Nikon, Japan) using a 10 × 20 lens, five representative visual fields with clearly stained myofibers were selected from each sample for imaging. The myofiber diameter, area, and density were measured using Image-Pro Plus software (Image-Pro Plus 6.0, Media Cybemetics, USA). Measurements followed the order of counting the top and not the bottom, and the left and not the right. The myofiber diameter was calculated as the average of the long and short diameters, while myofiber density was determined as the number of myofibers per unit area.

### 2.7 Composition of feedstuffs and longissimus thoracis muscle

Moisture (method 950.46), intramuscular fat (IMF; method 991.36), and ash (method 920.153) in the LM and feed samples were determined using the official methods of AOAC, Association of Official Analytical Chemists (2006). Nitrogen content was determined by the micro-Kjeldahl N method, and crude protein (CP) was calculated as nitrogen content times 6.25. IMF was quantified using an ANKOM^XT15^ extractor (ANKOM Technology, New York, USA). Feed samples were analyzed for neutral detergent fiber (NDF) and acid detergent fiber (ADF) contents using an ANKOM^200^ fiber analyzer (ANKOM Technology, New York, USA) according to the sequential detergent method of Van Soest et al. (1991). All measured parameters were determined in triplicate.

### 2.8 Fatty acids profiles

The LM samples and experimental diets were lyophilized (Scientz-10ND, Ningbo Scientz Biotechnology Co. Ltd., Ningbo, China) and ground to homogeneity. Fatty acid composition was analyzed in triplicate. For fatty acid analysis, IMF from the LM (0.2 g) was methylated directly following O'Fallon et al. (2007). The resulting fatty acid methyl esters (FAMEs) were extracted with 2 mL hexane containing methyl heneicosanoate internal standard (500 μg/mL, C21,0, 51535, Sigma Aldrich, USA) and 2 mL distilled water. After centrifugation (800 × *g*, 5 min), the hexane supernatant was collected in amber GC vials and stored at −20 °C.

FAMEs were analyzed using a gas chromatograph (Agilent technologies Trace 1300, Thermo Scientific, Santa Clara, CA, USA) equipped with a flame-ionization detector (FID) and an HP-88 fused silica capillary column (100 m × 0.25 mm inner diameter × 0.20 μm film thickness, Agilent Technologies, USA). For optimal separation of C18:1 isomers, the oven temperature was held isothermally at 180 °C for 70 min with nitrogen carrier gas (purity ≥99.99%) at a constant flow rate of 1.0 mL/min. Fatty acid profiles of the LM samples and experimental diets were identified by comparing retention time with commercially available FAME standards, including CMM47885 (37 FAME standards, Supelco, Bellefonte, PA, USA) and OBCFA standards (Mixture BR2, BR3 and Me 93; Larodan Fine Chemicals, Malmö, Sweden).

The health value of fatty acids was assessed based on the polyunsaturated fatty acid (PUFA):saturated fatty acid (SFA) ratio, the n-6:n-3 PUFA ratio, and the atherogenic (AI) and thrombogenic (TI) indices. AI and TI were calculated as (Ulbricht & Southgate, 1991):

$$\mathrm{AI}=\left[\left(C12:0\right)+\left(4\times C14:0\right)+\left(C16:0\right)\right]/\left(\sum \text{MUFA}+\sum \text{PUFA}\right);$$

$$\mathrm{TI}=\left[\left(C14:0\right)+\left(C16:0\right)+\left(C18:0\right)\right]/\left[0.5\times \sum \text{MUFA}+0.5\times \sum n-6\;\text{PUFA}+3\times \sum n-3\;\text{PUFA}+\left(n-3\right)/\left(n-6\right)\right];$$

where MUFA = monounsaturated fatty acids.

### 2.9 Oxidative capacity

Oxidative stability of the *longissimus* muscle was assessed by measuring total antioxidant capacity (T-AOC), superoxide dismutase (SOD) activity, malondialdehyde (MDA), and protein carbonyl (PC) content. Briefly, fresh muscle tissue (0.2 g) was homogenized in nine volumes of ice-cold saline (0.9% NaCl) to prepare a 10% (*w*/*v*) homogenate. The homogenate was centrifuged at 2500 × *g* for 15 min at 4 °C, and the resulting supernatant was collected for analysis.

All parameters were measured in triplicate using commercial assay kits (Nanjing Jiancheng Bioengineering Institute, Nanjing, China) according to the manufacturer's instructions. Absorbance was read on a microplate reader (BioTek Epoch 2, Agilent, Winooski, VT, USA). Data were normalized to the protein concentration of the supernatant, determined by a BCA protein assay kit (Beyotime Biotechnology, Shanghai, China), and expressed as U/mg protein for T-AOC and SOD, and nmol/mg protein for MDA and PC.

### 2.10 Statistical analyses

The experimental unit for all measurements was the individual yak. Data were analyzed using one-way analysis of variance (ANOVA) by SPSS software (version 21.0, SPSS Inc., Chicago, IL, USA), with fattening strategy (TG, LC and HC) considered as a fixed effect. Duncan's multiple comparison tests separated means where significance was detected among treatments. Significance was accepted at *P* < 0.05.

## 3 Results and discussion

### 3.1 Average daily gain and carcass traits

Concentrate supplementation enhanced growth performance, muscle deposition, and fat accumulation in feedlot compared to grazing yaks in the present study. Final body weight, average daily gain (ADG), hot carcass weight, and dressing percentage were greater (*P* < 0.01) in HC than LC and grazing yaks (Table 2), which aligned with previous studies (Frylinck et al., 2013; Koch et al., 2018; Liu et al., 2021), who reported that ADG and carcass weight were greater in feedlot than grazing steers. In addition, back fat thickness, *longissimus* muscle area, and marbling scores were greater (*P* < 0.05) in HC than grazing yaks, as was also reported by McIntyre et al. (2009). The poorer carcass traits in grazing than feedlot yaks in this study could be explained from two aspects. Grazing yaks expended more energy than feedlot yaks due to activities associated with foraging (Mwangi et al., 2019; Wicks et al., 2019), while HC group received diets rich in protein and energy which enhanced lean tissue accretion and adipose deposition and growth rate (Ladeira et al., 2016; Wicks et al., 2019).

**Table 2:** Effect of different fattening strategies on the carcass traits in yaks.

Items	Treatments 1			SEM ^2^	*P*-value
	TG	LC	HC		
Initial body weight, kg	213	211	224	3.9	0.371
Final body weight, kg	248^b^	260^b^	291^a^	5.7	˂0.01
Average daily gain, g/d	384^c^	548^b^	739^a^	37.8	<0.001
Hot carcass weight, kg	125^b^	131^b^	159^a^	4.0	<0.001
Dressing percentage, %	50.4^b^	50.7^b^	54.2^a^	0.44	<0.001
Back fat thickness, mm	1.67^c^	2.75^b^	4.14^a^	0.257	<0.001
*Longissimus* muscle area, cm^2^	49.9^b^	50.8^b^	53.3^a^	0.52	0.014
Marbling score	2.50^b^	2.42^b^	3.25^a^	0.258	0.027

^a,b,c^ Means within a row with different superscripts differ from each other (*P <* 0.05).

1 TG, traditional grazing; LC, low-concentrate stall-fed diet; HC, high-concentrate stall-fed diet.

2 SEM, standard error of the means.

ADG and back fat thickness were greater (*P* < 0.001) in the LC than TG yaks, but the other carcass traits did not differ between these two groups, which indicated that the performances of these two groups were similar. In the present study, the TG yaks grazed from June to September, coinciding with the peak biomass and nutritional quality of the grassland on the Tibetan Plateau (Guo et al., 2021). Therefore, when nutrient intake is adequate, grazing yaks can achieve body weight gain and carcass traits comparable to those of LC yaks. The back-fat thickness of yaks in this study ranged from 1.67 to 4.14 mm across the different management strategies, consistent with values reported by Kang et al. (2020) for feedlot yaks.

### 3.2 Meat quality attributes

Consumers are prioritizing health benefits and organoleptic-oriented quality when choosing meat products (Grunert, 2006; Resurreccion, 2004). Meat quality can be assessed by post-slaughter pH values, where pH_45min_ greater than 6.0 indicates high-quality meat, while values below 5.6 are associated with pale, soft, and exudative (PSE) meat. Conversely, pH_24h_ measurements above 6.0 are linked to dark, firm, and dry (DFD) meat (Lawrie & Ledward, 2006). In the current study, meat pH at 45 min and at 24 h did not differ among treatments and all groups fell within the range of high-quality meat. The meat colour indices, including L*, a*, b*, C*, and H*, also did not differ (*P* > 0.05; Table 3) among treatments. Meat colour and fat content are the most important characteristics that consumers consider when purchasing meat (Glitsch, 2000; Grunert, 1997). According to Ponnampalam et al. (2017), grazing animals often produce leaner carcasses due to nutritional deficiencies, which may affect pH and overall meat quality. Similar findings were reported by Gómez et al. (2021) and Frylinck et al. (2013), who noted variations in muscle pH_24h_ based on feeding regimes. However, in the present study, the pH_24h_ in grazing yaks did not increase, likely due to the high nutritional quality of the pasture during the peak grazing season. This aligned with other studies which reported no difference in ultimate pH between feeding systems (Apaoblaza et al., 2020; Nian et al., 2017).

**Table 3:** Effect of different fattening strategies on the meat quality in yaks.

Items	Treatments 1			SEM 3	*P*-value
	TG	LC	HC		
pH					
45 min	6.12	6.20	6.11	0.050	0.730
24 h	5.21	5.16	5.18	0.036	0.831
Meat colour					
L*_45 min_	34.7	34.8	35.1	0.09	0.205
a*_45 min_	18.4	18.5	18.6	0.04	0.131
b*_45 min_	8.10	8.03	8.19	0.037	0.213
C*_45 min_	20.1	20.2	20.4	0.04	0.067
H*_45 min_	23.7	23.4	23.7	0.10	0.506
WHC 2, %					
Drip loss	4.48^b^	4.88^a^	5.09^a^	0.095	<0.01
Cooking loss	33.8	37.5	36.1	0.98	0.329
Pressing loss	22.7	23.5	23.6	0.24	0.205
Shear force, N	101^a^	91.1^b^	78.6^c^	2.75	<0.001

^a,b,c^ Means within a row with different superscripts differ from each other (*P <* 0.05).

1 TG, traditional grazing; LC, low-concentrate stall-fed diet; HC, high-concentrate stall-fed diet.

2 WHC: water-holding capacity.

3 SEM, standard error of the means.

The WHC is a crucial characteristic of muscle, directly impacting economic losses in food production, as poor WHC can lead to the loss of water-soluble proteins, vitamins, and other nutrients (Huff-Lonergan & Lonergan, 2005). In the present study, drip loss was the only WHC parameter that differed (*P* < 0.01) between feedlot and grazing yaks, with greater drip loss in feedlot yaks (Table 3). This finding is consistent with the elevated MDA and PC concentrations in LC and HC meat (Table 7), indicating more oxidative damage. Structural modifications caused by lipid and protein oxidation can alter their functionality, thereby affecting product quality, including WHC. Zhang et al. (2013) reported that protein oxidation can inactivate or modify calpain activity, influencing fresh meat quality and protein degradation, ultimately leading to differences in muscle structure and drip loss. Additionally, Liu et al. (2010) reported that oxidative damage enlarges extracellular spaces between adjacent muscle fibers and disrupts the structural integrity of muscle cells, thereby reducing WHC.

In the present study, meat shear force ranged from 78.6 to 101 N and was greatest (*P* < 0.001) in TG yaks, intermediate in LC yaks, and lowest in HC yaks (Table 3).The shear force of yak meat in the present study was consistent with values reported for 4-year-old yaks (Xiong et al., 2021), but greater than those for beef steers (He et al., 2018). The feedlot yaks in the present study produced meat with lesser shear force and, therefore, was more tender than meat from grazing yaks. In addition, the shear force was lesser in the HC than LC yaks, and this can be attributed to several factors. The HC yaks received diets with high energy yield, promoting faster growth rates and fat deposition, including subcutaneous and IMF (Mwangi et al., 2019; Wicks et al., 2019). The greater back-fat thickness and IMF in HC yaks contributed to the lesser shear force than the grazing and LC yaks. These results were consistent with Gómez et al. (2021), who also reported that feedlot animals with faster growth rates produced more tender beef than grazing animals, and with Kang et al. (2020) and Liu et al. (2021) who reported that high-energy diets in feedlots reduced the shear force of yak meat.

The differences in yak meat tenderness in the present study were consistent with the lesser antioxidant capacity and more severe oxidation damage in feedlot yaks. The meat from feedlot yaks contained a lesser concentration of SOD and greater concentrations of MDA and PC than grazing yaks. Huff-Lonergan et al. (2010) reported that oxidative damage to proteins can induce physicochemical and structural changes, leading to myofibril breakage and reduced muscle cell integrity, ultimately enhancing tenderness. Brown et al. (2007) also noted that beef from feedlot steers tended to be more tender than from grazing steers. Furthermore, the greater fiber content in the diets of grazing than feedlot yaks may have contributed to reduced meat tenderness in the grazing yaks (Blank et al., 2017). In conclusion, the differences in meat tenderness in this study can be explained, at least partially, by variations in feed nutrient levels, animal body condition, and feeding management practices.

### 3.3 Histological observations

**Fig. 1:**
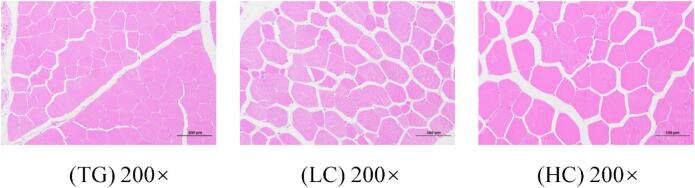
Morphology of *longissimus* muscle myofiber in yaks receiving different dietary intakes. TG, traditional grazing; LC, low-concentrate stall-fed diet; HC, high-concentrate stall-fed diet; 200 ×, 200 magnifications.

Currently, limited information is available on the effects of fattening strategies on the myofiber characteristics of yaks. Myofibers, the fundamental structural units of muscle, play a critical role in determining meat quality attributes such as tenderness, pH, colour, and drip loss (te Pas et al., 2004). Generally, smaller myofiber diameters are associated with lesser shear force and increased meat tenderness, while larger diameters are associated with tougher meat. Additionally, myofiber density is correlated negatively with myofiber diameter and area (Lepetit, 2007). The morphology of *longissimus* muscle myofibers were visualized in 200 × multiples by electron microscope, and the representative photos are presented in Fig. 1. HC yaks exhibited larger (*P* < 0.05) myofiber diameters and areas but lesser (*P* < 0.001) myofiber densities than the TG and LC yaks (Table 4), which can be attributed to the higher protein content and energy yield in the HC diet. The HC diet promoted faster growth rates than the TG and LC diets, leading to enhanced muscle deposition in the HC group (Ladeira et al., 2016; Wicks et al., 2019).

**Table 4:** Effect of different fattening strategies on the histological characteristics of *longissimus* muscle in yaks.

Items	Treatments 1			SEM 2	*P*-value
	TG	LC	HC		
Myofiber diameter, μm	59.0^b^	57.6^b^	67.7^a^	1.83	0.037
Myofiber density, N/mm^2^	527^a^	507^a^	343^b^	14.9	<0.001
Myofiber area, μm^2^	1949^b^	2006^b^	3003^a^	84.3	<0.001

^a,b^ Means within a row with different superscripts differ from each other (*P <* 0.05).

1 TG, traditional grazing; LC, low-concentrate stall-fed diet; HC, high-concentrate stall-fed diet.

2 SEM, standard error of the means.

### 3.4 Nutritional quality

Generally, the IMF content in beef cattle follows a trend similar to their growth performance, with greater IMF content in animals raised on concentrate-based diets than on forage-based diets. The HC diet, with its greater metabolizable energy yield, promoted greater fat deposition in muscle than in the TG and LC yaks (He et al., 2018), as evidenced by the greater (*P* < 0.05; Table 5) IMF content in the HC group. This aligns with findings by Aurousseau et al. (2004), who reported that animals fed concentrate diets had greater energy intake than those on pasture. Ruminants on forage produce mainly acetate and on concentrate produce mainly propionate, and adipocyte marble cells preferentially utilize glucose over acetate for fat synthesis (Rhoades et al., 2007), particularly in IMF adipocytes where fat synthesis is limited (Smith et al., 2009). Additionally, a greater IMF content enhances juiciness by increasing muscle moisture content, further improving meat tenderness. In the present study, the moisture content of *longissimus* muscle was greater (*P* < 0.01; Table 5) in feedlot (LC and HC) than grazing yaks indicating that the meat was more tender in the feedlot yaks. In addition, the shear force was greater (Table 3) in TG than feed-lot yaks, which also indicated that the meat from the stall-fed yaks was more tender than from the grazing yaks.

**Table 5:** Effect of different fattening strategies on the proximate composition of *longissimus* muscle in yaks.

Items (%)	Treatments 1			SEM 2	*P*-value
	TG	LC	HC		
Moisture	69.3^b^	71.0^a^	72.2^a^	0.39	˂0.01
Crude protein	24.1	24.9	25.4	0.27	0.153
Intramuscular fat	1.90^b^	1.94^b^	2.40^a^	0.095	0.047
Ash	1.67	1.70	1.75	0.016	0.124

^a,b^ Means within a row with different superscripts differ from each other (*P <* 0.05).

1 TG, traditional grazing; LC, low-concentrate stall-fed diet; HC, high-concentrate stall-fed diet.

2 SEM, standard error of the means.

Thus, it is reasonable to conclude that a high concentrate diet enhances dietary energy intake, leading to an increase in IMF and in meat tenderness. In contrast, pasture and low concentrate diets provide lesser energy and limit fat deposition, resulting in less tender meat. Differences in fattening management practices, animal body condition, and dietary nutrient levels likely contribute to the variations observed across studies.

### 3.5 Fatty acids profiles in meat

Owing to its importance in the human diet and health, the fatty acid profiles of meat have garnered increasing attention in recent decades. Fisher et al. (2000) highlighted that the healthiness of meat is associated closely with its fat content and fatty acid composition. The Food and Agriculture Organization (FAO) and the World Health Organization (2003) recommend reducing the intake of trans fatty acids (TFA) while increasing the consumption of PUFA. In the present study, meat from TG and HC yaks had greater (*P* < 0.01) PUFA contents and a more favorable PUFA: SFA ratio (*P* < 0.05) than LC yaks (Table 6). The PUFA:SFA ratio in yak meat (0.10–0.18) were consistent with values reported by Hu et al. (2021), but fell below the minimum recommended threshold of 0.4 (Wood et al., 2008). Wood et al. (2008) also noted that forage-based diets promoted greater levels of PUFA content in ruminant meat than concentrate-based diets. Forage-based diets provide primarily acetate, which contributes less than 20% of the acetyl units involved in intramuscular fatty acid biosynthesis, whereas concentrate-based diets promote propionate production, accounting for approximately 70% of the acetyl units (Smith et al., 2009). Conversely, excessive SFA intake increases blood low-density lipoprotein (LDL) cholesterol concentrations, thereby increasing the risk of cardiovascular diseases (Siri-Tarino et al., 2010). In the present study, meat from TG yaks had greater (*P* < 0.001) SFA and lesser (*P* < 0.01) MUFA contents than HC yaks, consistent with findings that SFA and MUFA are incorporated preferentially into triglycerides with increased IMF deposition (Wood et al., 2008).

**Table 6:** Effect of different fattening strategies on the fatty acid profiles of *longissimus* muscle in yaks (g/100 g of total identified fatty acid methyl esters).

Items	Treatments 1			SEM 3	*P*-value
	TG	LC	HC		
Saturated fatty acid (SFA)					
C10:0	0.11	0.11	0.11	0.004	0.986
anteiso-C13:0	1.82	1.79	1.81	0.129	0.998
isoC14:0	1.88	1.83	1.81	0.123	0.971
C14:0	2.55	2.41	2.52	0.109	0.883
anteiso-C15:0	0.18^a^	0.14^b^	0.12^c^	0.008	<0.001
C15:0	0.10^b^	0.11^b^	0.17^a^	0.014	0.036
isoC16:0	0.96	0.82	1.17	0.080	0.207
C16:0	23.2	25.1	22.5	0.55	0.136
C17:0	0.70^a^	0.72^a^	0.55^b^	0.020	<0.001
isoC18:0	0.09^b^	0.16^a^	0.14^a^	0.009	<0.001
C18:0	28.4^a^	26.6^ab^	25.4^b^	0.49	0.026
C20:0	0.30^a^	0.26^b^	0.18^c^	0.013	<0.001
C22:0	0.28	0.31	0.25	0.015	0.175
C23:0	2.19	1.86	2.22	0.138	0.543
C24:0	0.05^a^	0.03^b^	0.02^b^	0.004	<0.001
Monounsaturated fatty acid (MUFA)					
C14:1	0.34	0.26	0.41	0.039	0.355
C16:1	0.61^b^	0.68^a^	0.58^b^	0.015	<0.01
C17:1	0.46^ab^	0.52^a^	0.43^b^	0.016	0.032
C18:1n9t	3.36^a^	1.17^b^	1.07^b^	0.264	<0.001
C18:1n9c	21.6^b^	27.8^a^	27.5^a^	0.85	<0.001
C18:1	1.40^b^	1.36^b^	1.95^a^	0.080	<0.001
C20:1n9	0.08^b^	0.12^a^	0.13^a^	0.007	<0.001
C24:1n9	0.06	0.04	0.06	0.005	0.180
Polyunsaturated fatty acid (PUFA)					
C18:2	0.66^a^	0.44^b^	0.45^b^	0.032	<0.001
C18:2n6t	0.19^a^	0.05^b^	0.03^c^	0.018	<0.001
C18:2n6c	5.03^b^	3.96^b^	7.58^a^	0.506	<0.01
C18:3n6	0.04	0.03	0.05	0.004	0.311
C18:3n3	1.99^a^	0.63^b^	0.26^c^	0.192	<0.001
C20:2	0.05	0.05	0.06	0.003	0.252
C20:3n6	0.29	0.25	0.33	0.019	0.218
C22:2n6	0.06^a^	0.03^b^	0.01^b^	0.006	<0.001
C20:5n3	0.81^a^	0.34^b^	0.18^b^	0.073	<0.001
C22:6n3	0.13^a^	0.03^b^	0.03^b^	0.013	<0.001
Summary 2					
SFA	62.8^a^	62.2^a^	58.9^b^	0.52	<0.001
MUFA	28.0^b^	32.0^a^	32.1^a^	0.69	<0.01
PUFA	11.2^a^	6.44^b^	9.23^a^	0.679	<0.01
PUFA: SFA	0.18^a^	0.10^b^	0.16^a^	0.011	0.012
n-3 PUFA	2.92^a^	1.01^b^	0.47^b^	0.275	<0.001
n-6 PUFA	5.60^b^	4.32^b^	7.99^a^	0.520	<0.01
n-6: n-3 PUFA	1.93^c^	4.30^b^	17.1^a^	1.091	<0.001
AI	0.90	0.92	0.79	0.030	0.168
TI	2.10^b^	2.53^a^	2.35^ab^	0.063	<0.01

^a,b,c^ Means within a row with different superscripts differ from each other (*P <* 0.05).

1 TG, traditional grazing; LC, low-concentrate stall-fed diet; HC, high-concentrate stall-fed diet.

2 AI, atherogenic index; TI, thrombogenic index.

3 SEM, standard error of the means.

**Table 7:** Effect of different fattening strategies on the oxidative stability of *longissimus* muscle in yaks.

Parameter in fresh muscle 2	Treatments 1			SEM 3	*P*-value
	TG	LC	HC		
T-AOC, U/mg	0.36	0.35	0.36	0.010	0.996
SOD, U/mg	5.33^a^	3.97^b^	4.32^b^	0.181	<0.001
MDA, nmol/mg	0.14^c^	0.36^a^	0.23^b^	0.028	<0.001
PC, nmol/mg	2.34^b^	6.64^a^	2.70^b^	0.608	<0.001

^a,b,c^ Means within a row with different superscripts differ from each other (*P <* 0.05).

1 TG, traditional grazing; LC, low-concentrate stall-fed diet; HC, high-concentrate stall-fed diet.

2 T-AOC, total antioxidative capacity; SOD, superoxide dismutase; MDA, malondialdehyde; PC, protein carbonyl.

3 SEM, standard error of the means.

Meat from TG yaks contained greater (*P* < 0.01) levels of antiso-C15:0, C20:0, C24:0, C18:1n9t, C18:2, C18:2n6t, C18:3n3, C22:2n6, C20:5n3, and C22:6n3, but lesser (*P* < 0.01) levels of isoC18:0, C18:1n9c, and C20:1n9 than feedlot yaks. This difference can be attributed to the fatty acid profiles of the ingested diets, as pasture contains less MUFA and more PUFA than concentrate diets (Guo et al., 2012). Smith et al. (2006) reported that palmitic acid (C16:0) and stearic acid (C18:0) can be converted to palmitoleic acid (C16:1) and oleic acid (C18:1) *via* delta-9 desaturase activity. The greater (*P* < 0.001) C18:1 and lesser (*P* = 0.026) C18:0 levels in HC yaks indicated an enhanced delta-9 desaturase activity and fat anabolism (Wood et al., 2008), consistent with the greater fat content in HC meat (Table 5). Several studies demonstrated that oleic acid accounted for close to 40% of the total MUFA content, with a beneficial effect on humans (Daley et al., 2010; Terés et al., 2008). Thus, high-concentrate diets can contribute positively to oleic acid levels in yak meat.

Of particular interest are n-3 PUFAs, owing to their reputed disease-preventive and therapeutic properties. (Mozaffarian & Wu, 2011). Concentration of n-3 PUFA was greater (*P* < 0.001) in grazing than feedlot yaks mainly due to elevated levels of α-linolenic acid (ALA, C18:3n3), eicosapentaenoic acid (EPA, C20:5n3), and docosahexaenoic acid (DHA, C22:6n3) in TG yaks. The high ALA content in pasture could explain the greater ALA levels in meat in grazing than feedlot yaks (Fisher et al., 2000). ALA serves as a precursor for EPA and DHA, which are crucial for foetal development and cardiovascular health (Swanson et al., 2012). In the present study, meat from HC yaks had greater (*P* < 0.01) n-6 PUFA levels than TG and LC yaks; n-6 PUFA levels are associated with various diseases and body fat accumulation and human obesity (Muhlhausler & Ailhaud, 2013; Simopoulos, 2004). The n-6:n-3 PUFA ratio was greatest (*P* < 0.001) in HC yaks, intermediate in LC yaks and lowest in grazing yaks. A high n-6:n-3 ratio can lead to thrombosis and inflammation, and, in turn, atherosclerosis, obesity, and diabetes (Kimura et al., 2019). While most foods have n-6:n-3 ratios between 10:1 and 30:1 (Simopoulos, 2011; Valencak et al., 2015), the British Department of Health (1994) recommends a ratio below 4 for optimal human health. Only the grazing yak meat met this standard in the present study, underscoring its potential health benefits.

The AI and TI of meat are used widely to evaluate the health implications of fatty acid profiles, particularly risks of atherosclerosis and coronary thrombosis (Folch et al., 1957). An AI below 1 is considered acceptable for human health (Knock, 2007) and the meat of all groups was below this threshold. The grazing yaks had a lesser (*P* < 0.01) TI than LC yaks, suggesting that the meat from the grazing yaks was less likely to cause coronary thrombosis.

### 3.6 Oxidative capacity in meat

Oxidative stability is a critical factor influencing meat shelf life (Kochhar & Henry, 2009). The antioxidant capacity of meat depends on genetic factors (breed), nutritional composition (diet), and pre-slaughter handling conditions. Feeding strategies and diets affect the oxidative stability of meat by altering the balance between antioxidant compounds and readily oxidized substrates, such as highly unsaturated fatty acids, in muscle tissue (Cherif et al., 2018). The concentration of SOD, a key antioxidant enzyme that neutralizes free radicals, was greater (*P* < 0.001) in grazing than feedlot (LC and HC) yaks, potentially enhancing the antioxidant capacity of the meat. Fresh alpine forage is rich in antioxidant nutrients such as vitamin E, vitamin A, carotenoids, amino acids, and fatty acids, which could explain the elevated SOD levels in meat from grazing yaks (Guo et al., 2012; Prache et al., 2003; Röhrle et al., 2011). These nutrients not only improve antioxidant capacity but also contribute to producing healthy animal products.

MDA concentration, a marker of lipid oxidation, and PC, an indicator of protein oxidative modification, are used commonly to assess oxidative damage. In the present study, MDA and PC concentrations were lesser (*P* < 0.001) in TG than LC and HC yaks, likely due to differences in management practices, concentrate content, and diet quality among the treatments (Guo et al., 2012; Prache et al., 2003; Röhrle et al., 2011). These findings indicate that the antioxidant capacity of grazing yaks was stronger than feedlot yaks, potentially reducing oxidative damage and improving meat quality and shelf life (Descalzo & Sancho, 2008). Consistent with this premise, Santé-Lhoutellier et al. (2008) reported greater PC levels in lambs fed concentrate diets than grazing lambs.

Interestingly, HC yaks had lesser (*P* < 0.05) MDA and PC concentrations than LC yaks, which may be linked to their growth performance. However, this contrasts with findings by He et al. (2018), who reported greater MDA and PC levels in steers fed high-concentrate diets than those on high-forage diets, suggesting that greater forage intake enhances antioxidant capacity. Further studies are warranted to determine the mechanisms responsible for these variations in antioxidant capacity.

### 3.7 Limitations and future perspectives

This study provided a comparative evaluation of different fattening strategies on yak meat quality, yet several limitations should be noted. Although the current sample size was deemed adequate and aligns with prior studies on this species, a larger sample size in future studies would provide a more solid basis for generalizing the findings. Furthermore, the evaluation was restricted to the *longissimus* muscle; a more comprehensive assessment would also examine other major muscles. Moreover, the yaks grazed during the peak pasture season, and outcomes may vary under winter or transitional conditions, a crucial consideration for year-round production systems. The observed differences in meat quality were likely influenced by the overall dietary matrix beyond individual nutrients, highlighting the need for future research to elucidate these complex interactions. Moving forward, studies should aim to optimize the balance between meat yield, organoleptic characteristics, economic benefits, and health-promoting qualities to better align production with consumer demands.

## 4 Conclusions

Fattening strategies influenced carcass traits, muscle and fat deposition, and key meat quality parameters in yaks. A high concentrate diet was the primary factor enhancing carcass traits and reducing shear force in beef, while also playing a major role in shaping fatty acid profiles and nutritional quality. However, feedlot yaks fed low- and high-concentrate diets had lesser antioxidant capacity and were more susceptible to oxidative damage than grazing yaks. This oxidative stress likely contributed to greater drip loss in muscle. Meat from grazing yaks was less tender but healthier than feedlot yaks due to its favorable PUFA profile.

## CRediT authorship contribution statement

**Zhiyuan Ma:** Writing – original draft, Validation, Software, Investigation, Formal analysis, Conceptualization. **Hongshan Liu:** Validation, Investigation. **Abraham Allan Degen:** Writing – review & editing. **Lintong Zhang:** Validation, Investigation. **Jiandui Mi:** Writing – review & editing. **Jianwei Zhou:** Writing – review & editing, Supervision, Methodology, Investigation, Funding acquisition, Formal analysis, Conceptualization.

## Declaration of competing interest

The authors declare that they have no known competing financial interests or personal relationships that could have appeared to influence the work reported in this paper.

## Data Availability

Data will be made available on request.
